# A discourse analysis of social inequities, gender, and stigma in tuberculosis policies of seven countries from Africa, Asia, Europe and South America

**DOI:** 10.1080/16549716.2025.2547150

**Published:** 2025-09-05

**Authors:** Mariana Valdivino, Alethe de Vaulgrenant, Klasien Horstman, Yuliya Chorna, Ruby Stein, Jeremiah Chikovore, Amrita Daftary, Nora Engel

**Affiliations:** a Department of Health, Ethics & Society, Care and Public Health Research Institute CAPHRI, Faculty of Health, Medicine and Life Sciences, Maastricht University, Maastricht, The Netherlands; b School of Global Health and Dahdaleh Institute of Global Health Research, York University, Toronto, Canada; c Public Health, Societies and Belonging Division, Human Sciences Research Council, Durban, South Africa; d Centre for the AIDS Programme of Research in South Africa (CAPRISA), Nelson Mandela School of Medicine, University of KwaZulu-Natal, Durban, South Africa; e Athena Institute, Faculty of Science, Vrije Universiteit Amsterdam, Amsterdam, The Netherlands

**Keywords:** discourse analysis, qualitative methods, TB policy, policy analysis, social drivers of disease

## Abstract

**Background:**

Interventions tackling the social aspects of tuberculosis (TB) are widely suggested, yet we miss insights into how policies incorporate these. The language and framing of policies to address TB can lend important insights into how these social drivers are perceived, problematized, and responded to.

**Objective:**

To understand how discourses in current TB policies frame social dimensions of TB, especially concepts of social inequity, gender, and stigma.

**Methods:**

We conducted a comparative critical discourse analysis of twenty-one publicly available TB-related policies from Belarus, Brazil, Indonesia, Mozambique, Netherlands, Portugal, and Romania, countries with diverse epidemiological, geographical and sociopolitical contexts. Documents were sourced from public websites from May – September 2024. The Bacchi approach was used to analyze policy framings of social inequities, gender, and stigma.

**Result:**

While policies from Brazil and Indonesia showed greater attention to social inequities, gender, and stigma, and were more explicitly reflective of an equity-oriented and people-centered approach, overall, a dominant biomedical perspective was observed that individualizes responsibility for cure. This tends to disregard issues of social inequity, obscures gender relationships and the multiple dimensions of stigma. At the same time, allocation of individual as well as structural responsibility for TB risk and outcomes co-existed.

**Conclusions:**

Explicit and implicit discourses about TB within health-related policies can influence the nature of attention given to the social dimensions of TB and can shape corresponding responses to the disease. We recommend a participative policy process that includes a broader set of actors to ensure documents are responsive to social realities.

## Paper Context

- **Main findings**: The discourses used within tuberculosis policy documents have the potential to shift the focus of issues between different stakeholders or reduce the urgency to address a problem.
- **Added knowledge**: This study comparatively analyzed policy documents from seven different countries and uncovered a dominant biomedical perspective which might overshadow the complexity of the biopsychosocial needs of persons affected by tuberculosis.
- **Global health impact for policy and action**: Policymakers and various stakeholders should be mindful of how documents frame tuberculosis, affected communities, and care efforts.

## Background

Tuberculosis (TB), the world’s oldest infectious disease, is preventable and curable. However, the global burden of TB has stayed remarkably high [1]. TB is thought to have returned to the leading cause of death from a single infectious agent (in place of COVID-19) [1]. In 2023, an estimated 10.8 million people fell ill with TB and 1.25 million deaths were attributed to TB globally [1]. TB is considered a social disease, disproportionately affecting people in low- and middle-income countries, and globally, in communities facing resource shortages and systemic social disadvantages. Current global plans to end TB emphasize attention to addressing social inequities including those that are gender-related, as well as stigma, thus recognizing them as fundamental barriers to ending TB [2–9]. Prevention, treatment and care measures accordingly demand transdisciplinary interventions that address the social, economic, and environmental drivers of TB [10,11].

Despite the broad global recognition for an equitable TB response that addresses social inequities, gender, and stigma, the TB policy, programming, research, and funding arenas continue to maintain a dominant focus on the medical aspects of TB – centered on preventing disease spread through infection control measures (such as isolation, quarantine, contact tracing, and public surveillance) and technological interventions (such as tests, drugs, and treatment monitoring). Important progress has been made in the countries researched with regard to the numbers of people treated and diagnosed. To illustrate, the treatment success rate in new and relapse cases in Mozambique was estimated to be 95% in 2022 [1] compared to 87% in 2015 [12]. Yet, interview-based studies of people’s lived experiences with TB reveal a wide array of socioeconomic and gender-related barriers to TB health-seeking and adherence to interventions in both high and low TB incidence settings [13–20]. Further improvements will pivot on finding the large number of undiagnosed people with TB, better preventing and addressing complications including drug resistance and HIV co-infection, which in turn requires social inequities, gender, and stigma to be tackled. That rates of TB remain high suggests a need to revisit the assumptions and values that underlie the current measures and interventions, and examine how social and structural dimensions of TB, particularly social inequity, gender, and stigma, are being woven into policies within TB-affected countries. Analyzing these together allows to discern how their combined and intersecting nature is being addressed in policy documents.

Policies, typically articulated through documents, and the language used within them, have the power to influence perceptions of a problem among stakeholders, the urgency to act, and the forms of action taken [21,22]. Policies serve as guiding schemata for programming decisions and practices at various levels. Policies, a key output of governments, are central to the organization, functioning, and governance of contemporary societies [23]. They shape the way publicly pertinent issues (for example, health, welfare, law, labour) get defined and how key stakeholders become categorized or given roles and characterization (for example, *‘subject’, ‘citizen’, professional’, ‘criminal’*) [23]. Policies thereby carry implied assumptions, hidden in language, about the problems and solutions they seek to address. Depending on how problems are framed within policies, different actors who are responsible for identifying solutions, may be led to seek differential remedial strategies and adopt differential degrees of urgency to act [21,24].

Notably missing in the TB literature to date is a critical analysis of guidelines and frameworks that govern TB caregiving and *‘control’* measures. In response to this gap, we undertook a critical discourse analysis of TB policy texts to examine how the related challenges of social inequities, gender, and stigma are conceptualized, problematized and addressed in policy spheres at the country-level [25]. Analyzing how TB is framed, and how social inequities, gender, and stigma in TB are conceptualized and addressed at national policy levels can help understand how and why particular solutions are opted for, and how responsibility to operationalize solutions is assigned [26]. Discourses of TB within national policies are crucial in framing action on the social drivers of TB. Analyzing TB from a national policy level in both high and low incidence countries may not only help identify neglected gaps around its social dimensions, but can shed insights of how these dimensions may be addressed [27].

### Social inequities and TB

Health inequities are ‘*differences in health that are unnecessary, avoidable, unfair, and unjust’* [28]. They are rooted in social inequities that bar access to and enjoyment of equal rights and opportunities for people facing social disadvantage [29]. Social science scholars have long implicated broad social and political processes and movements such as colonialism, globalization, migration, forced displacement, growing and rapid urbanization as key factors contributing to the shape of the global TB epidemic. These processes create(d) ripe conditions for the spread of TB: crowded living often in barely habitable conditions, unemployment or employment with low wages and salaries, malnutrition, precarious migrant status, social and economic deprivation in general, incarceration, poor access to health care, and poor social protections [30,31]. People affected by TB often also face other forms of social disadvantage related to the different yet intersecting identities they carry, including race, gender identities and sex, ethnicity, class, and country of origin [32–34]. TB compounds these systemic disadvantages and mounts new instabilities such as loss of socioeconomic productivity, stigma, and discrimination, gendered vulnerabilities, and increased demands for public spending by governments [33,34]. Today, TB is normalized as a disease affecting marginalized populations at the intersection of complex health and social risks, and the quintessential disease of social inequity [19,31]. It is important to mention that the term used here onwards – responsibility for TB outcomes – is defined as the patterns of diagnosis, treatment success, and prevention efficacy, that are deeply influenced by social inequities.

### Gender and TB

Physiological and biological differences between sexes explain part of the risk and vulnerability to TB and its sequelae [35,36]. Socially constructed gender roles and expectations exert strong influences on the behaviors and circumstances of people who may be affected by TB, from *‘patients’* to care providers, and further influence risk of and vulnerability to the disease. These biosocial factors explain some of the differences in health status, probability of developing TB disease, and experiences of illness and related outcomes encountered by people of different genders. Differences in rates of TB have been linked to gender-attributed particularities in the frequency and level of exposure to the TB bacteria, heteronormative behaviors and circumstances that can heighten the risk of infection, disease development and poor clinical outcomes (see Table 1 for data on the gender disparity in the countries researched in 2023) [25–28].

**Table 1.** Country epidemiological data [1,37].

	TB prevalence per 100 000 population in 2023	Total estimated TB incidence in 2023	TB incidence rate per 100 000 population 2023	People diagnosed with new and relapse case of TB 2023	% women new and relapse TB case notification (aged ≥15 years)	% men new and relapse TB case notification (aged ≥15 years)	% children new and relapse TB case notification (0–14 years)
Brazil	57	103 000 (87 000–119 000)	49 (41–56)	92 185	29%	68%	3%
Indonesia	591	1 090 000 (995 000–1 210 000)	387 (354–432)	804 836	34%	49%	17%
Mozambique	368	121 000 (74 000–181 000)	361 (220–537)	116 317	42%	46%	12%
Netherlands	5	820 (700– 940)	4.5 (3.9–5.2)	710	35%	62%	3%
Portugal	19	1 600 (1 400–1 900)	16 (13–18)	1 501	30%	67%	3%
Romania	65	10 000 (9 000–12 000)	55 (47–63)	9 122	27%	68%	5%
Belarus	42	2 500 (1 900–3 200)	27 (21–35)	1 555	23%	76%	1%

Epidemiological data suggests worse outcomes for men than for women, necessitating a gender-responsive approach to TB that is deliberately inclusive of men [33,38]. Concurrently, a focus on women is crucial given the distinct implications of TB on their health, and gender roles and expectations. Both women, who comprise most household caregivers and the frontline health workforce, and men, who comprise most of the migrant, mining, mobile workforce, and prison population, face occupational and social risks for TB [33,34]. Women face added risks during pregnancy and post-partum, increasing antenatal and postnatal morbidity and mortality [39]; more often neglect their own needs, delay TB treatment, and face financial barriers to navigating care on account of less agency and economic capital; and within health facilities, women can also have their complaints more easily dismissed by providers [40,41]. Men, on the other hand, are placed in occupational and social situations that increase their use of tobacco, alcohol, and other drugs, and place them at greater risk for poor outcomes in TB. They too can neglect seeking care – despite their relatively greater agency – to safeguard economic productivity, earning income, and their socially defined gender identities, roles, and expectations. Within health facilities, they too can receive care that is inattentive to their social and occupational obligations [33]. Social inequity and stigma thus interface with gender roles and expectations, affecting both women and men in relation to TB. People of non-binary gender identities can face added forms of inequity and discrimination. The nuanced ways by which the social dimensions of TB are encountered by people of diverse genders are thus a key consideration for TB policies.

### Stigma and TB

Stigma is a social process characterized by exclusion, rejection, blame or devaluation, that results from experience, perception, or reasonable anticipation of an adverse social judgement about a person or group [42,43]. Stigma is considered a major bottleneck to TB elimination [5,27,44] and reviewing policy level interventions has been identified as a critical gap [45].

In its various forms (such as anticipated, perceived, enacted, and internalized), and through various actors (such as partners, family members, community members, health care workers and even practices ingrained within health systems), stigma imposes a multitude of impacts on people affected by TB. These consequences include self-isolation, guilt, shame, denial, psychological effects, delayed care seeking and poor adherence including to preventive and treatment measures, the fear of and loss of relationships, neglect, discrimination, and other human rights violations such as verbal and physical abuse, denial of legal status, and exclusion from work or school. These have a significant impact on the physical and mental health and wellbeing of people affected by TB, at times affecting well-meaning caregivers and care providers [44].

This policy discourse analysis aimed to examine how the related challenges of social inequity, gender, and stigma are being conceptualized and addressed in the language of policy texts from different countries around the world.

## Methods

### Study design

We carried out a critical discourse analysis (CDA) [46] of publicly available TB policy texts from seven countries – Belarus, Brazil, Indonesia, Mozambique, the Netherlands, Portugal, and Romania, purposively selected, in the first instance, on the basis of their diverse TB epidemiological profiles, geographical and sociopolitical context and, in the second instance, study team members’ fluency in some of the relevant languages, specifically Portuguese, Dutch and Russian.

Mozambique (Sub Saharan Africa) and Brazil (South America) are countries with high prevalence of TB, concentrated among people living with HIV and socioeconomically vulnerable populations, particularly people experiencing homeliness, children, people deprived of their liberty and indigenous populations (in the case of Brazil). The countries share a language and were both colonies of Portugal. Indonesia is an Asian country with a high TB and MDR-TB burden, with challenges related to access to healthcare among low-income populations and a former colony of the Netherlands, but linguistically different. Romania and Belarus are Eastern European countries with high prevalence of TB, (as well as MDR-TB in the case of Belarus) concentrated among socioeconomically vulnerable populations, including people with substance use disorder and people that have been deprived of their liberty. Netherlands (Western Europe) and Portugal (Southern Europe) are countries with low incidence of TB, concentrated among people with a history of migration and/or living in situations of homelessness, reflecting structural and social vulnerability.

The findings may be of value for countries with similar epidemiological, geographical and sociopolitical diversity as well as for global TB policies.

### Theoretical approach

Bacchi’s (2009) [26] approach to policy analysis, centered on the question *‘What’s the Problem Represented to Be’* (WPR) offers a useful framework for CDA. It contrasts with models that envision solutions on the basis of taking a given policy problem for granted, and instead brings the nature or definition of the problem itself into question [1,26,47]. Accordingly, our analysis was guided by six questions: 1) What is the problem of TB represented to be in the policies examined? 2) What presuppositions or assumptions underline this representation of the problem of TB? 3) How has this presentation come about? 4) What is left unproblematic in this problem representation, where are the silences, and can the ‘*problem’* be thought about differently? 5) What effects are produced in this representation? 6) Lastly, how/where is this representation produced, disseminated, and defended, and how could it be questioned, disrupted, and replaced? Based on this, we could understand how TB is being perceived, problematized, and responded to in each country, identify overlaps and distinctions, and uncover how implicit and explicit discourses emergent within policy texts can shape national responses to TB [48].

### Data sources

For each country, we included publicly accessible policy documents that were enacted at the time of analysis. These spanned a period between 2011 and 2024. We started with the main guideline for TB control and included additional policy documents that were referenced with regard to aspects relating to social inequity, gender and stigma.

Subsequent searches were carried out using ‘*tuberculosis’* and ‘*TB management’* as search terms for the websites of ministries of health (for Brazil, Indonesia, Mozambique, the Netherlands, and Portugal), the StopTB Partnership through the *Portal Legistiv* (for Romania), and for (publicly available) reports on the Belarus healthcare system (for Belarus).

### Data collection and analysis

Documents were analyzed in their original language (for Belarus, Brazil, Mozambique, the Netherlands, and Portugal) or translated using software (*DeepL*) and checked for accuracy (for Indonesian, and Romanian). In keeping in line with methodological guidance for discourse analysis [46,49–51], this involved re-reading the policies multiple times and writing memos and reflective notes. In doing so, the following questions, adapted from the WPR approach [26], helped to guide our critical review of TB policy texts, in relation to framings of the problem of TB, and framings and attentiveness to social inequities, gender, and stigma as they apply to TB (see Appendix A for the complete data extraction and analysis guide):

- What relationship is portrayed between TB and social inequities, gender, and stigma?
- What social inequities are exposed or concealed?
- How is gender portrayed?
- How is stigma considered and framed?
- How do the countries compare in their framings of TB, and more specifically, framings of social inequities, gender, and stigma?
- What are the potential strengths, gaps, and opportunities to address or intervene on social inequities, gender and stigma related to TB in each country, and across countries?
- What considerations (gaps and issues) can be highlighted in future policies and related interventions?

## Results

In total, 21 policy documents were analyzed, including 11 national TB program reports or TB control (management) strategies, seven clinical guidelines, and three resolutions or laws (see Table 2). Three documents focused on TB and HIV, and one on drug-resistant TB. Documents were labelled with an alphanumeric code comprising country letter/s and a sequential number. The analysis led us to explicate four major findings, as elaborated ahead (see Appendix B for complete tabulated findings).

**Table 2.** List of analyzed policy documents (intended audience is defined as follows: TB program/policy managers (who do not directly interact with patients), healthcare workers (who interact with patients), patients, wider society (non-patients).

Document	Stakeholders Involved	Intended Audience	Language of Document/Language of Analysis	Code
Order No 15 of 13 January 2014 on Guidelines on Organization of Work in Tuberculosis Infection foci and identification of contact cases, MOH Belarus, 2014 [52]	Minister of Health (MoH)	Managers	Russian/Russian	BE1
Clinical Protocol: Diagnosis and treatment of patients with tuberculosis (adults, child, populations), MOH Belarus, 2019 [53]	MoH	Managers	Russian/Russian	BE2
Governmental Program on National health and demographic security for 2021–2025, Council of Ministers of Belarus, 2021 [54]	Council of Ministers	Managers	Russian/Russian	BE3
Brazil Free of Tuberculosis – National Plan to End Tuberculosis as a Public Health Problem (strategies for 2021–2025) [55]	MoH; Health Surveillance Department; Department of Chronic Conditions Diseases and Sexually Transmitted Infections; and external collaboration (regional stakeholders)	Managers	Portuguese/Portuguese	BR1
Manual of Recommendations for Tuberculosis Control in Brazil 2019 [56]	MoH; Health Surveillance Department; Department of Chronic Conditions Diseases and Sexually Transmitted Infections	HealthcareProfessionals	Portuguese/Portuguese	BR2
Resolution nº444 of July 6, MOH Brazil, 2011 [57]	National health council, and MoH	Not specified	Portuguese/Portuguese	BR3
National Strategy for Tuberculosis Control in Indonesia 2020–2024 [58]	Multidisciplinary report – different ministries; external partners (e.g. USAID); and other different sectors and groups involved	Managers	Indonesian/English	I1
Guiding for TB/HIV Collaborative Activities, MOH Mozambique, 2019 [59]	MoH; National Directorate of Public Health; National Tuberculosis Control Program; National Council for the Fight against AIDS; National Council for the Control of STIHIV/SIDA and, external partners (e.g. USAID, I-TECH, etc.)	Healthcare Professionals	Portuguese/Portuguese	M1
Evaluation and Management of TB Patient, MOH Mozambique, 2019 [60]	MoH; National Directorate of Public Health; National Tuberculosis Control Program; I-TECH Mozambique; The Aurum Institute	Managers and Healthcare Professionals	Portuguese/Portuguese	M2
Tuberculosis Guide, National Institute for Public Health and Environment (RIVM), Netherlands [61]	RIVM, MoH	Healthcareprofessionals	Dutch/Dutch	N1
National Tuberculosis Control Plan 2016–2020, Netherlands [62]	Representatives from RIVM, KNCV, Dutch Thoracic Society (NVALT), the Netherlands Tuberculosis Control Policy Committee (CPT) Association of Tuberculosis Control Physicians ((VvAwT), the Dutch Association for Medical Microbiology (NVMM), the Dutch Nursing Society (V&VN) and the Medical Technicians’ Association (MTMBeVe).	Not specified	Dutch/Dutch	N2
National Tuberculosis Control Plan Update 2021–2025, Netherlands [63]	Representatives from: TB survivors; RIVM; CPT; Steering Committee mandated DPGs; KNCV; LAMO; NVALT; NVMM; NVIB; RAC’ers; REC managers; RTCs; V&VN; VvAwT.	Not Specified	Dutch/Dutch	N3
National Program for HIV Infection, SIDA and Tuberculosis, MOH Portugal, 2017 [64]	National Program for HIV Infection, AIDS and Tuberculosis; MoH; Directorate-General for Health	Not specified	Portuguese/Portuguese	P1
Report on TB Surveillance and Monitoring in Portugal, MOH Portugal, 2021 [65]	MoH, Directorate-General for Health	Not specified	Portuguese/Portuguese	P2
Tuberculosis in Portugal – Challenges and Strategies, MOH Portugal, 2018 [66]	MoH, Directorate-General for Health	Not specified	Portuguese/Portuguese	P3
National Tuberculosis Control Strategy in Romania 2015–2020, Working Group of Romania, 2015 [67]	Working group comprising representatives of the MoH, the National Tuberculosis Control Program, WHO, and other government institutions and NGOs	Managers	Romanian/English	R1
Methodological Guidelines of 21 September 2015 for the implementation of the national tuberculosis prevention, surveillance, and control programme, MOH Romania, 2015 [68]	MoH	Healthcare Professionals	Romanian/English	R2
Law No 302 of 10 December 2018 on Tuberculosis control Measures, Parliament of Romania, 2018 [69]	Parliament of Romania	Managers	Romanian/English	R3
Methodological guidelines of 4 January 2018 on the control of tuberculosis transmission in pneumo-phthisiology health units and other institutions at risk in Romania, MOH Romania, 2018 [70]	MoH	Managers and Healthcare Professionals	Romanian/English	R4
National Guideline of 5 October 2020 Management of chemo-resistant tuberculosis cases, MOH Romania, 2020 [71]	MoH	Healthcare Professionals	Romanian/English	R5
National Tuberculosis Control Strategy in Romania − 2022–2030, 2022 [72]	Global Fund, WHO staff and consultants, and national experts	Managers	Romanian/English	R6

### Definition of TB

A medical framing of TB is evident across the documents. TB is defined as a disease stemming from an infectious agent by all the seven countries. Documents from Brazil additionally characterize TB as a public health threat and a chronic condition with strong social determination and a barrier to socio-economic development (BR1, BR3). Such a framing can emphasize clinical measures over socio-economic interventions and can implicate greater responsibility to healthcare providers over others working in social sectors where TB and risk of TB may be determined.

### Social inequities

This section explores how vulnerability to TB and responsibility for TB outcomes are conceptualized within different policy contexts (see also Table 3). ‘*Vulnerability’* to TB or being ‘*high-risk*’ for TB are concepts commonly used in the policy documents to draw attention to inequities in TB; with slight variations based on a country’s epidemiological profile, groups of people considered ‘similar’ are then assigned to these categories. These groups are people living with HIV, people deprived of their liberty (prisoners), people experiencing homelessness (homeless persons), people who have migrated from high burden countries (immigrants, refugees), people who use alcohol and other drugs, pregnant women, and children. Statistical data on TB diagnosis, treatment, and prevention is typically shared to portray disease patterns within countries, and to identify groups that experience higher rates of TB and characterize their vulnerability or risk.

**Table 3.** Example quotes illustrating discourse on social inequity.

	Belarus	Brazil	Indonesia	Mozambique	Netherlands	Portugal	Romania
IndividualResponsibility	‘[…] *patient motivation is critical* […]’ (B1, p. 17)	–	–	[one exclusion criterion for preventive therapy for TB for children and teenagers is] ‘*alcohol/drug abuse; bad adherence*’ (M1, p. 28)	‘*Approximately 20% of new immigrants to the Netherlands […] have LTBIs. That prevalence correlates to the incidence in the countries of origin.*’ (N2, p. 24)	‘[a limited number of vulnerable patients] *who will not resort to health* *care by choice’*’ (P1, p. 6)	‘*non-adherence is the patient’s refusal or inability* *to take prescribed medication as directed. This behavior is the biggest problem in TB control and can have serious consequences*’ (R2, p. 22)
StructuralResponsibility	‘[…] *carrying out measures to strengthen the treatment* *of patients with tuberculosis through the introduction of the state social order in the provision* *of tuberculosis care in the regions*.’ (BE3, p. 12)	‘*Reducing the harm caused by the harmful use of alcohol and other substances or minimizing the environmental impact of overcrowded prisons are just as important as the therapeutic regimen.’* (BR1, p. 188)	‘*the current social protection is insufficient to mitigate the socioeconomic impact of tuberculosis*’ (I1, 84)	*‘Where/when is not possible to test all patients with GeneXpert, this cases should be prioritized: […]’* (M2, p13).	–	‘*structure dedicated to TB* *currently has fewer professionals allocated and trained* […]’ (P1, p. 11)	‘[the country’s rural population] *often at higher risk of exposure due to their living situation and at higher risk of developing* *active disease due to poor health status* […] *Overall, the rural population is proportionately affected by TB and is more* *likely to experience treatment failure and drop-out*’ (R1, p. 11)
Interventions for ‘*High-Risk*’ Groups	‘*increasing the availability* *of prevention, diagnosis and treatment of tuberculosis for vulnerable groups of the population* […]’ (BE3, p. 11)	‘*Basic social protection* […] *It is aimed at people living in vulnerable situations due to poverty, lack of income*, … .’ (BR2, p. 303)	‘*The implementation of the* *TemPO strategy should be extended to inpatient services by targeting screening and* *assessment to all hospital patients with respiratory complaints, patients at risk of TB* […]’ (I1, 76)	*‘Therefore*, [healthcare] *services should screen all patients in the waiting room using cough officers* […]’ (M1, p. 33)	‘*To achieve these objectives the screening for latent infection and preventive treatment of risk* *groups, such as new immigrants and asylum-seekers will be enforced*’ (N2, p. 9)	*‘[…] given the epidemiological evolution of tuberculosis in Portugal, only children with individual or community risk factors for tuberculosis were vaccinated*. {with BCG]’ (P3, p. 5)	‘*screen*[ing of] *all inmates for TB symptoms upon entry into the prison and refer*[al of] *TB* *suspects for screening with rapid diagnostic methods*’ (R1, p. 55)
Social/Economic Support Measures	‘[The healthcare professionals are also responsible to inform their patients about] *the possibility of receiving social and material assistance from the state and public organizations*’ (BE2, p. 13)	‘*When health services identify tuberculosis patients in a situation of vulnerability, they can advise them to seek social assistance services* […] *and access benefits.’* (BR2, 303–304)	‘*Strengthening support services and psychosocial protection for TB patients*. […] *There is a need for support services and social protection for people reported as tuberculosis cases*’ (I1, p. 84)	‘*Ensure at least one counseling session before starting TB treatment*’ (M1, p. 52)	–	–	‘[with TB patients being] *entitled to leave and compensation for temporary* *incapacity for work, without conditions of contribution period, for the entire period of* *treatment, until they are cured*’ (R3, p. 4)
Coercive/Punitive-like Measures	‘*involuntary hospitalisation and treatment of patients with TB who evade treatment if all other* *approaches to providing treatment for RR-TB are used* ’ (BE2, p. 11)	–	–	‘*The patients with bad* *adherence to treatment must not be offered TPI until* *membership is secures’* (M2, p. 124)	–	–	‘*failure to comply with the obligations laid down in paragraph 1 shall be deemed* *to be a break of the following (1) by the patient diagnosed with tuberculosis shall entail the* *suspension of the monthly maintenance allowance*’ (R3, p. 4)

Attention to social inequity also emerges in how responsibility for TB outcomes is attributed. In many documents, responsibility is assigned to individual-level behaviors and decisions, with scarce acknowledgement of the underlying social processes amidst which vulnerability may be perpetuated and/or decisions are made. For example, documents from the Netherlands allocate responsibility for TB acquisition to people’s immigrant or asylum-seeking status (N1, N2, N3). In Portugal, there is an understanding that ‘vulnerable patients’ make active choices to avoid health care (P1, p. 6).

That said, alongside an individualist framing, most documents also acknowledge the role of wider social factors and place responsibility for TB outcomes on actors beyond individuals with TB. In Belarus, for example, responsibility for TB acquisition is assigned to poor individual habits, though recognizing these are propagated by broader social and cultural practices: ‘[there is need to] *fight against bad habits, provided that the general population is involved in physical culture, tourism, and cultural events, and the need to raise the level of education’* (BE3, Article 10). In Mozambique, treatment adherence is held to be the individual’s responsibility, although it is acknowledged that people’s social conditions play a role (M1). Some of the documents, such as from Belarus, Brazil, Indonesia, Mozambique Romania, and Portugal, draw attention to the enabling and/or disabling role of health system attributes – equipment and (better) methods for TB diagnosis and/or treatment (BE1, M1, M2), DOTS (R1) See Table 3), decentralization (I1), and improvements at the level of health care facilities (R1) and providers (BR1, P1) – in the achievements of TB infection control and elimination. Romania, for example, maintains that high-risk facilities should be responsible for instituting measures to reduce transmission (R1), and Brazil suggests health care providers should be sensitive to non-medical barriers to TB treatment (BR1).

These tensions, of emphasizing individual as well as structural responsibility, extended to the measures suggested for ensuring prevention and access to treatment. Documents from Portugal, Belarus, Brazil, and Mozambique emphasize interventions among people considered to be at high-risk for TB, including newborns and children, professionals employed in high-risk jobs, close contacts of people with TB disease, and/or people living with HIV; although, interventions are limited to vaccines and/or TB preventive treatment (BR2, M2, P3, BE1). Other documents, including from Brazil, Belarus, Indonesia and Romania, portray broader interventions that may alleviate inequities, such as social, psychosocial, educational (see also section Stigma), and material assistance including nutrition and transportation (BR2, I1, M1, BE3, R2). For example, Brazil emphasizes that people living with TB should maintain balanced diets through ‘*popular restaurants, community kitchens and food banks*’ (B2, p. 304–305). Overall, documents from Indonesia and Brazil most visibly portray policy targets to tackle social inequities in people affected by TB (I1, BR1, BR2, BR3). For example, in Indonesia ‘*the national Tuberculosis programme has developed a patient and community feedback mechanism’* (I1, p. 53) in order to increase community participation; while Brazil wants to address deficits in social protection, by including TB patients on local social programs (BR2, p. 302). At least three documents, despite acknowledging the role of social factors in earlier sections, proceed to recommend what seem to be coercive and/or punitive-like measures for people who are non-adherent which is likely to exacerbate inequities. For example, Belarus has a policy of involuntarily hospitalizing them through court mandates (BE2) and Romania outlines removing their social support and pursuing legal action if they knowingly infect other people (R5). Mozambique recommends for children and adolescents with a history of poor adherence, or a record of alcohol or drug use to be excluded from the programme (M2).

### Gender

Gender-related vocabulary, limited to the terms women or females and men or males, is used as part of an epidemiological and statistical framing to report on TB outcomes within documents from Belarus, Indonesia, Portugal, and Romania and one of the Brazilian documents (BR2) (see also Table 4). The vocabulary is used to portray differences in outcomes on the basis of biological sex rather than social identities or roles. Consistent with reported global trends [1], in all countries TB incidence is noted to be higher in men or males (see Table 2). Brazil (BR2), Mozambique, Portugal, and Romania specify men or males as being at higher risk for TB, while Belarus target only woman as a risk group. Indonesia does not explicitly define either men or women. The Netherlands stops at alluding to men’s intersectional risks as a result of their higher representation among other high-risk populations such as immigrants, asylum seekers, and detainees; in general, the country’s policies highlight the importance of focusing on TB interventions among refugees and asylum seekers, the demography of which is predominantly male, but without mentioning gender specific interventions explicitly (N1, N2, N3). In all documents, no reference is made either to non-binary persons, or interventions that address risk factors and vulnerabilities, improve outcomes, or tackle TB broadly, especially among men.

**Table 4.** Example quotes illustrating discourse on gender.

		Belarus	Brazil	Indonesia	Mozambique	Netherlands	Portugal	Romania
Biological Sex	Male	–	*‘PDL* [people deprived of their liberty] *are mostly from the segments of the population most affected by TB: young men* […] *(BR2*, p. *233)*	‘*The proportion who did not seek treatment was higher in males*.’ (I1, 61)	*‘New ART starts in adult patients (men and women).’* (M1, p. 40)	–	*‘Male predominance continues: for every three women diagnosed there were seven* *men diagnosed in 2016 (ratio of 2.7).’* (P1, p. 5)	*‘TB affects men more than women, with men accounting for 69% of all cases in* *2012.’* (R2, p. 11)
	Female	*‘All women of reproductive age with TB are strongly advised to use contraception to prevent pregnancy* […]’ (BE2, p. 19)	*‘The treatment of TB, besides being important for the pregnant woman’s condition*, […]’ (BR2, p. 112)	‘[…] *stigma is more prevalent in women than men* […]’ (I1, 82)	*‘Who should be screened for TB at the health facility?* […] *pregnant women* […]’ (M2, p. 5)	–	*‘Of the total number of infection cases recorded in 2020, 1353 were female (47.3%).’* (P2, p. 29)	*‘TB affects men more than women, with men accounting for 69% of all cases in* *2012.’* (R2, p. 11)
Social Identities		–	–	–	–	–	–	–
Other Identities		–	*‘person with TB’* (BR2, p. 37)	*‘people with TB’* (I1, p. 24)	–	–	–	*‘people diagnosed with tuberculosis’* (R3, p. 3)

The Brazilian, Mozambican, and Dutch documents call attention to women and/or pregnant women as being at risk for TB. Documents from Belarus, and Mozambique focus considerably on TB prevention and treatment in women of reproductive age and pregnant women, specifically to safeguard childbearing women’s reproductive health as well as fetal and infant health (BE1, M1, M2). Mozambique highlights ‘*maternal death’* as a complication from the use of TB drugs among HIV-positive pregnant woman, alongside fetal outcomes such as ‘*miscarriages, intrauterine deaths, prematurity, and low birth weigh*t’ (M1, p. 27–28). The importance of focusing on pregnant women and women of reproductive age was represented to be due to the impact on the fetus (in the case of Mozambique, MI and M2), and the effect on the fertility rate within the country, considered one of the indicators of failures of the state program in Belarus ‘*All women of reproductive age with TB are strongly advised to use contraception to prevent pregnancy* *… If this is ineffective, at the beginning of treatment it is necessary to use barrier methods of contraception until the amount decreases adverse reactions to anti-tuberculosis drugs, after which oral contraceptives can be re-assigned to the patient’* (B2, p. 87).

### Stigma

Most policies, with the exception of Belarus, Mozambique and the Netherlands, explicitly mentioned stigma (see also Table 5). Stigma was, however, not defined in any of the documents, suggesting it held a universal, presumed meaning within and across the documents. A few documents touched upon stigma drivers and impacts. Policies from Brazil, for example, stated a need to ‘e*liminate the stigma and discrimination associated with certain population groups, which have historically been barriers to public policy*.’ (BR2, p. 41), acknowledging there were key populations affected by TB that faced systemic barriers and were more susceptible to stigmatization. Policies from Indonesia also recognized that stigma affected health-seeking behaviors (I1).

**Table 5.** Example quotes illustrating discourse on stigma.

		Belarus	Brazil	Indonesia	Mozambique	Netherlands	Portugal	Romania
Contains explicitly the term stigma		–	*‘eliminate the stigma* […]’ (BR2, p. 41)	“*stigma amongst patients, families* *and staff still exists*” (I1, p. 82)	–	–	‘[…] *fighting stigma and discrimination*.’ (P1, p. 10)	‘[…] *avoiding stigmatisation and social marginalisation* […]’ (R2, p. 22)
Defines stigma		–	–	–	–	–	–	–
Drivers of stigma and/or impact		*“communicate and inform the public when a* *significant ‘outbreak’ of tuberculosis infection is detected, in order to reduce the negative* *impact of distorted messages in the media”* (BE1, p. 13)	‘e*liminate the stigma and discrimination associated with certain population groups, which have historically been barriers to public policy*.’ (BR2, p. 41)	*“A 2018 study by the Global Fund showed that stigma and discrimination* *from health workers impacted motivation to use services.”* (I1, p. 84)	–	*”It was noted that the epidemiology of tuberculosis in the* *Netherlands is significantly influenced by the numbers and origins of migrants and* *asylum-seekers arriving in the country.”* (N2, p. 9–10)	–	–
Measures against stigma	Education	*“teach[ing] the patient [a set of] rules … [to] improve his general sanitary* *and medical literacy and form a strong motivation for the strict implementation of all rules and* *recommendation*.” (BE1, p. 16)	‘*12. guarantee the production and broadcasting of Prevention, Education and Awareness campaigns about mass TB on a permanent basis.’* (BR3)	*“Develop communication strategies for counselling and stigma reduction of drug- sensitive tuberculosis, drug-resistant tuberculosis and TB-HIV. […] Staff ability to communicate effectively needs to be improved by including IEC promotion and effective TB-HIV communication materials in staff training.”* (I1, p. 82)	–	–	*‘Process of improvement in Tuberculosis literacy in the population and in health professionals’* (P2, p. 31)	“[…] *publish TB educational material in newspapers, magazines; to present radio broadcasts on* *the same theme, to introduce and expand the broadcasting of short films or commercials on* *several TV channels with the message of correct TB detection and treatment* […]” (R2, p. 22)
	Psychosocial	“[…] *emotional support and psychological assistance … every time the patient visits an* *anti-tuberculosis dispensary”*, [this is done to avoid the patient feeling] *“rejected* [and] *unwilling to communicate.”* (BE2, p. 13)	–	–	–	–	–	“[…] *support patients through psychological support*, *charity, avoiding stigmatisation and social marginalisation and creating a real chance for full* *compliance.”* (R2, p. 22)
	Material Support	‘[…] *organization of video-controlled* […]’ [treatment of TB] (BE3, p. 62)	–	–	–	–	–	–
	Community Engagement	–	–	*“Reduction of* *stigma and discrimination needs to be done at the community level, workplace and health* *care facilities*. (I1, p. 82)	–	–	–	–
Stigmatizing language		*‘Epidemiological threat’* (BE1, p. 4)	–	‘*Develop TB suspect referral tools* […]’ [I1, p. 196]	*‘infected’* (M2, p. 3)	–	–	‘*Identification of TB suspects*’ [R1 p. 3].

Despite the absence of a definition of stigma, several documents did propose measures to address TB stigma. The policies of Belarus, Brazil, Indonesia, Portugal, and Romania suggested educational and media campaigns to reduce stigma (I1, BR1, BR2, B3, P2, BE1, R2, R3), and policies of Belarus and Romania went so far as to also recommend psychosocial services and anti-discrimination training for healthcare professionals (BE1, R2, R3). The *‘emotional support and psychological assistance … every time the patient visits an anti-tuberculosis dispensary’, is suggested to avoid the patient feeling ‘rejected [and] unwilling to communicate’* (BE2). Additionally, Belarus’ policy recommends material and social support during treatment to avoid disrupting normal life routines, for instance through provision of equipment for video-observed treatment (BE2). Indonesia’s policy included community engagement as a means to reduce stigma (I1).

Notwithstanding some direct attention to stigma, most of the documents also contained terms that are stigmatizing, even after robust translation checks [22]. Documents from Belarus, Indonesia, Mozambique, and Romania used terms such as ‘*infected’* (e.g. M2, p. 3) or ‘*suspects’* (I1, R1, R2, R3, R4, BE1, BE2) to describe people living with TB (See Appendix B).

The individualization of TB risk and recovery also bred degrees of negative stereotyping and labelling. The policies of Belarus, Mozambique, Netherlands, Portugal, and Romania all cast responsibility for cure on the person living with TB, with Belarus, Mozambique, and Romania also using stigmatizing terms as stated earlier (M2, P1, N1, N2, N3, R1, R2, BE1, BE2).

## Discussion

This study uses critical discourse analysis to assess how the related challenges of social inequities, gender and stigma are conceptualized and addressed in national TB policy documents of seven countries. It finds that policies differ with regard to how they frame and address the social dimensions of TB. Policies from Brazil and Indonesia showed greater attention to social inequities, gender, and stigma. Recommendations for TB prevention, treatment, and care were more explicitly reflective of an equity-oriented and people-centered approach. By contrast, policies from Belarus, Mozambique, Netherlands, Portugal, and Romania engender a largely bio-medicalized framing of TB. In what follows we discuss detailed findings for social inequity, gender and stigma.

The policies analyzed framed social inequities as a statistical epidemiological factor related to TB risk and clinical outcomes, as opposed to a complex social construct that mediates pathways of risk and recovery and manifests in different ways across populations, settings or within a given country. The available research literature argues collectively for the importance of alleviating social inequities for advancing in the fight against TB [32,73–76]. Socially disadvantaged groups and risk groups for TB are virtually identical, with the disease disproportionately affecting people in low- and middle-income countries, communities facing resource shortages, socially disadvantaged people, and those deprived of their liberty. Yet, only the documents from Indonesia, Brazil, and Belarus included clear policy targets to address social inequities through social protection and prioritizing equity in healthcare. In line with this framing of social inequity, the analysed policies mostly located responsibility for TB at either the individual or structural level, while in some documents both forms of responsibility co-existed. It was not surprising then, that in the documents from Brazil and Indonesia, responsibility for TB treatment and its containment was allocated at the health system level. In the policies of Portugal and the Netherlands, an emphasis on risk groups was accompanied with individualizing responsibility for TB treatment and its containment, without discussing the underlying social inequities influencing behavior. Placing responsibility for TB on the individual or on specific risk groups accentuates social inequities and their sequelae [77]. Interestingly, in the policies of Mozambique, Belarus, and Romania, responsibilities for TB treatment and its containment were allocated both at the level of individuals as well as at the wider health system. This tension, of emphasizing individual and structural responsibility, extended to the measures suggested for ensuring access to treatment: strong reliance on DOT or punitive-like measures for non-adherence (which may further increase social inequities) co-existed with measures to enhance access to treatment and social support.

According to our findings, indications of a multi-sectorial approach to address social inequity were only found in a few documents. Therefore, opportunities do exist to strengthen addressing social inequities in TB through a biosocial approach, which extends beyond the medicalization of TB to target strengthening health systems and decreasing social inequities within society as a whole [77]. The analyzed policies, importantly, do allocate some responsibility with the health system, particularly through facilitating access to treatment and social support, as in the policies of Brazil, Indonesia, Belarus, and Romania. However, addressing social inequities requires a multi-sectorial approach (for instance through addressing poor and overcrowded living conditions) [5]. If TB policies insufficiently address the multi-sectorial nature of social inequity, they risk not meeting the needs and enabling access to care for disadvantaged groups, which ultimately carry the main risk for TB.

The analyzed documents use either medical definitions or gender-neutral language to conceptualize gender which both can suppress the relationship between TB and gender and may lead to underrepresentation and unwanted exclusions. The conceptualization of gender in the policies encompassing a medical definition use vocabulary associated with males and females. Nevertheless, the documents of Brazil, the Netherlands, and Romania use gender-neutral language, which may signify the suppression of the relationship between gender and TB according to their country’s contexts. Contrastingly, epidemiological evidence is not provided within the Belarusian, Indonesian, and Mozambican policies, but considerable focus on women of reproductive age suggests conception of an epidemiological relationship between gender and TB. Ultimately, gender agnostic TB policies, that insufficiently address the diverse gender identities, roles, expectations and relations underlying TB emergence and TB outcomes, can fail to meet the diverse needs of those who are affected by TB in their population.

There appears to be many opportunities to strengthen attention to stigma, and its connectedness to gender and social inequities, in the policy texts reviewed. Whereas several country texts acknowledge stigma, and make recommendations on strategies to reduce stigma, a clear definition of what causes stigma in those settings and the impacts of stigma are not discussed. The lack of consistent definitions of stigma has also been noted for the available literature on TB stigma reduction interventions [45]. Most of the recommendations to reduce stigma therefore lacked the requisite multidimensionality, which may include community engagement, counselling, social protection, human rights approaches, and peer support. Following the Language Matters guidance developed by Stop TB Partnership alongside TB affected communities [78], the WHO has phased out stigmatizing terms in its policy guidance. While particular terms may continue to have familiarity and relevance at the country level, and within the health system, there is an opportunity for decisionmakers to mimic ongoing efforts in other settings and at the global level, to transform the words and phrases used to describe people affected by TB and use it as a launching pad for a more equitable and respectful response to TB.

Overall, our findings show that these differences in policy framing may be better explained by broader political, institutional, and socio-historical contexts than by TB epidemiology alone. No clear pattern emerges when examining our study findings according to country context. For example, Brazil and Mozambique take very different approaches to equity and stigma, despite both being high TB-HIV burden settings. Likewise, countries with similar epidemiological profiles (e.g. Portugal and Romania) vary in how they address gender and stigma. Documents also differ with regard to intended audience and stakeholders involved in writing process. But also here, no clear pattern emerges. The identified discourse did not change depending on whether the document was intended for healthcare workers in the form of technical guidelines or multisectorial stakeholders in the form of a strategic national plan. With the exception of Romanian and two Portuguese policies (which were developed by the ministry of health or their council), the remaining policies were written as part of larger multidisciplinary groups including ministry of health, regional stakeholders, other relevant health departments, and/or external partners. The analysis underlines how even though many ideas, data and technologies about TB are globally shared, national policy infrastructures still play a major role in the construction of TB and what communities affected by TB in different parts of the world can expect. This is similar to what professionals and citizens in border-regions experience when national infrastructures pull them back and toward national issues, as happened during COVID-19, preventing them from their normal mobility and benefitting of different governance regimes [79]. It suggests that there are some limits for global action and that attention to infrastructural work at the level of national systems is required. Future research is needed to attend to the broader political, institutional, and socio-historical contexts within which policy gets written.

Strengths of this study include the comparative analysis across diverse countries, guided by an explicit framework offering a structured way to discourse analysis and that most of the documents were analyzed in their original language, which allowed the researcher to follow its peculiarities.

We also note several limitations. A limitation is that the analysis is limited to policies that we were able to access via public websites, which could mean we did not exhaustively access the entire policy infrastructure of a country, and our findings might therefore present a limited picture. Moreover, the Indonesian and Romanian policies needed to be translated prior to analysis, and it is possible some meanings were lost during translation. In addition, the study focuses on policies as a data source, and more comprehensive context-specific reviews including additional document research and interviews with key stakeholders could further illuminate how the policies were developed, are being understood, and also being implemented.

## Conclusion

Human rights, stigma, and gender are emerging as key components of the global TB response [5]. Partially in response to advocacy efforts of TB affected communities and civil societies and important political moments leading to the United Nations High-Level Meetings on TB [4]. This comparative discourse analysis examined how social inequities, gender, and stigma are addressed in national TB policies.

Policies influence the national discourse on TB through the writing, framing, and addressing of targets and outcomes. Within the policies reviewed, a dominant biomedical perspective was observed that individualizes responsibility for cure and tends to disregard issues of social inequity. Consequently, many policies revealed considerable gaps with respect to key dimensions of social determinants of TB. Gender was conceptualized in biomedical or gender-neutral terms. Stigma was mentioned but not defined. Recommendations to address social inequity, gender and stigma therefore lacked requisite multidimensionality and multi-sectoriality. At the same time, in several policies, allocation of individual as well as structural responsibility for TB risk and outcomes co-existed. This tension extended to the measures suggested for ensuring prevention and access to treatment. A key implication of our findings is that countries in reasonably similar circumstances could give greater consideration to how policies are framed and written. Beyond technical and biomedical considerations, this could entail a more participatory policy process that includes a broader set of actors attuned to the different social drivers of TB, including people with TB and survivors, activists, communities, multi-sectoral actors, and others. Doing so can help ensure that policies are more responsive to social realities and better positioned to address the root causes of TB. Overall, clear definitions and descriptions of how stigma, social inequities, and gender disparities affect TB outcomes could be woven into TB policies. This would help avoiding unwanted exclusions and arriving at recommendations that invite multidimensional and multi-sectorial action to address these social drivers of TB.

Future research should examine the broader political, institutional, and socio-historical context of policy development, how the TB discourse within health policies aligns with other social sector policies and how discourses on social inequities, gender, and stigma are realized in practices.

## Supplementary Material

SM3_COREQ_checklist.docx

SM1 and 2 merged_revised.docx

## Data Availability

All data were sourced from public documents which are referenced in the reference list.
