# Supplementary material for: A discourse analysis of social inequities, gender, and stigma in tuberculosis policies of seven countries from Africa, Asia, Europe and South America
Source: Glob Health Action. 2025 Sep 5;18(1):2547150. doi: 10.1080/16549716.2025.2547150 (PMC12416019; doi:10.1080/16549716.2025.2547150)
Supplement: SM3_COREQ_checklist.docx [file ZGHA_A_2547150_SM2931.docx]

**SM3 - Consolidated criteria for reporting qualitative studies (COREQ): 32-item checklist**

| **No.** | **Item** | **Description** | **Section** |
| --- | --- | --- | --- |
| **Domain 1: Research team and reflexivity** | | | |
| Personal characteristics | | | |
| *1.* | Interviewer/facilitator | Which author/s conducted the interview or focus group? | N/A |
| *2.* | Credentials | What were the researcher's credentials? *E.g. PhD, MD* | N/A |
| *3.* | Occupation | What was their occupation at the time of the study? | N/A |
| *4.* | Gender | Was the researcher male or female? | N/A |
| *5.* | Experience and  training | What experience or training did the researcher have? | N/A |
| Relationship with participants | | | |
| *6.* | Relationship  established | Was a relationship established prior to study commencement? | N/A |
| *7.* | Participant knowledge of the interviewer | What did the participants know about the researcher? *E.g. Personal goals, reasons for doing the research* | N/A |
| *8.* | Interviewer  characteristics | What characteristics were reported about the interviewer/facilitator? *E.g. Bias, assumptions, reasons and interests in the research topic* | N/A |
| **Domain 2: Study design** | | | |
| Theoretical framework | | | |
| *9.* | Methodological  orientation and theory | What methodological orientation was stated to underpin the study? *E.g. grounded theory, discourse analysis, ethnography,*  *phenomenology, content analysis* | Discourse analysis see study design p.9 |
| Participant selection | | | |
| *10.* | Sampling | How were participants selected? *E.g. purposive, convenience, consecutive, snowball* | N/A |
| *11.* | Method of approach | How were participants approached? *E.g. face to-face, telephone, mail, email* | N/A |
| *12.* | Sample size | How many participants were in the study? | N/A |
| *13.* | Non-participation | How many people refused to participate or dropped out? What were the reasons for this? | N/A |
| Setting | | | |
| *14.* | Setting of data  collection | Where was the data collected? *E.g. home, clinic, workplace* | N/A |
| *15.* | Presence of non  participants | Was anyone else present besides the  participants and researchers? | N/A |

| *16.* | Description of sample | What are the important characteristics of the sample? *E.g. demographic data, date* | N/A |
| --- | --- | --- | --- |
| Data collection | | | |
| *17.* | Interview guide | Were questions, prompts, guides provided by the authors? Was it pilot tested? | See p.10 - 12 |
| *18.* | Repeat interviews | Were repeat interviews carried out? If yes, how many? | N/A |
| *19.* | Audio/visual recording | Did the research use audio or visual recording to collect the data? | N/A |
| *20.* | Field notes | Were field notes made during and/or after the interview or focus group? | N/A |
| *21.* | Duration | What was the duration of the interviews or focus group? | N/A |
| *22.* | Data saturation | Was data saturation discussed? | N/A |
| *23.* | Transcripts returned | Were transcripts returned to participants for comment and/or correction? | N/A |
| **Domain 3: analysis and findings** | | | |
| Data analysis | | | |
| *24.* | Number of data  coders | How many data coders coded the data? | Two. See p. 24, authors contributions |
| *25.* | Description of the  coding tree | Did authors provide a description of the coding tree? | Yes. See supplementary material 2 |
| *26.* | Derivation of themes | Were themes identified in advance or derived from the data? | Themes were identified in advance through the WPR approach. See p. 11-12 |
| *27.* | Software | What software, if applicable, was used to manage the data? | DeepL was used for translation of some documents and highlighted on p. 11 |
| *28.* | Participant checking | Did participants provide feedback on the findings? | N/A |
| Reporting | | | |
| *29.* | Quotations presented | Were participant quotations presented to illustrate the themes / findings? Was each quotation identified? E.g. Participant number | Quotations were provided throughout the results to illustrate findings. See p. 15-20 |
| *30.* | Data and findings  consistent | Was there consistency between the data presented and the findings? | Yes. Information in suppl material table 2 and the results are consistent |
| *31.* | Clarity of major  themes | Were major themes clearly presented in the findings? | Yes. The findings were discussed under three main themes See. p. 15-20 |
| *32.* | Clarity of minor  themes | Is there a description of diverse cases or discussion of minor themes? | N/A |
