# Supplementary material for: A discourse analysis of social inequities, gender, and stigma in tuberculosis policies of seven countries from Africa, Asia, Europe and South America
Source: Glob Health Action. 2025 Sep 5;18(1):2547150. doi: 10.1080/16549716.2025.2547150 (PMC12416019; doi:10.1080/16549716.2025.2547150)
Supplement: SM1 and 2 merged_revised.docx [file ZGHA_A_2547150_SM2930.docx]

**Supplementary Material:**

SM 1 Appendix A Topic Guide Based on the WPR Approach

SM 2 Appendix B Analysis Based on the WPR Approach

SM 3: Coreq checklist

**SM1: Appendix A Topic Guide Based on the WPR Approach**

**Gender**

Question 1 *What’s the ‘problem’ represented to be in a specific policy or policy proposal?*

· Is the word gender (in Portuguese the translation would be sex/gender) mentioned?

· If yes, what is the meaning of gender in the text? (male/female, diverse sexual identities)

· In which ways is TB related to gender? (epidemiologically, socially, as a determinant, or in terms of stigmatization)

· How is this relationship represented?

· How has this relationship arisen? To what extent is this relationship significant in the document?

Question 2 and Question 3 *What presuppositions or assumptions underpin this representation of the ‘problem’? and How has this representation of the ‘problem’ come about?*

· What gender assumptions can be derived from the policy and its relationship with TB?

· Does the policy explain the different effects of TB on the different genders?

· Is gender underrepresented because of higher incidence in the male population? (or vice-versa)

· Is there enough literature on both sexes to be possible to have equal gender representation recommendations? How does this affect the relationship between TB and gender?

· Based on the introduction of the relationship between gender and TB, how has this relationship been developed within the policy? How is it progressing throughout the text?

Question 4 *What is left unproblematic in this problem representation? Where are the silences?*

· What representation of gender is being used in the policy?

· Is someone being excluded because of the gender definition provided?

· Are gender-neutral/inclusive terms applied?

Question 5 *Can the ‘problem’ be thought about differently?*

· What are the effects that gender has on the problematization of TB?

· How is gender used to describe the outcomes (target populations)?

· Does the use of gender for the problematization of TB cause unintended stigmatization or exclusion?

Question 6 *What effects are produced by this representation of the ‘problem’?*

· Was the group that produced this document equally distributed in gender terms?

· Would the inclusion of gender-neutral/inclusive terms enable the policy to target a wider population affected by TB?

· Are the outcomes of the policy gender specific?

**Social Inequities**

Question 1 *What’s the ‘problem’ represented to be in a specific policy or policy proposal?*

· Are social inequities (income, healthcare, and social classes) mentioned?

· Are they defined? If so, how?

· How are they related to TB? (epidemiologically, socially, as a determinant, or in terms of stigmatization)

· How is this relationship represented?

· How has this relationship arisen? To what extent is this relationship significant in the documents?.

Question 2 and Question 3 *What presuppositions or assumptions underpin this representation of the ‘problem’? and How has this representation of the ‘problem’ come about?*

· What assumptions on social inequities can be derived from the policy and its relationship with TB?

· How is TB represented in terms of social inequities (income, healthcare, and social classes)? Does the policy explain the different effects of TB on the different social statuses?

· Are social inequities underrepresented because of higher incidence in a specific social group?

· Is there enough literature on social inequities to ensure a holistic representation of social inequities and therefore recommendations? How does this affect the relationship between TB and social inequities?

· Based on the introduction of the relationship between social inequities and TB, how has this relationship been developed within the policy? How is it progressing throughout the text?

· Is the incidence higher (related to these social groups) to be addressed in the documents?

Question 4 *What is left unproblematic in this problem representation? Where are the silences?*

· If there is a definition, is someone being excluded because of it? Does the definition include all groups?

Question 5 *Can the ‘problem’ be thought about differently?*

· What are the effects of social inequities have on the problematization of TB?

· How are social inequities used to describe the outcomes (target populations)?

Question 6 *What effects are produced by this representation of the ‘problem’?*

· Would the inclusion of social groups enable the policy to target a wider population affected by TB?

· Are the outcomes of the policy specific to social inequities?

**Stigma**

Question 1 *What’s the ‘problem’ represented to be in a specific policy or policy proposal?*

· Is the word stigma mentioned?

· What is the meaning of stigma in the text?

· In which ways is TB related to stigma?

· Do they use stigmatizing language? Do they address recurrently particular groups with this language?

Question 2 and Question 3 *What presuppositions or assumptions underpin this representation of the ‘problem’? and How has this representation of the ‘problem’ come about?*

· What assumptions regarding stigma can be derived from the policy and its relationship with TB?

· How is TB represented in terms of stigma?

· Does the policy explain the different effects of TB?

· Is TB-related stigma underrepresented in low-incidence countries?

· Based on the introduction of the relationship between stigma and TB, how has this relationship been developed within the policy? How is it progressing throughout the text?

Question 4 *What is left unproblematic in this problem representation? Where are the silences?*

· If there is a definition provided for ‘stigma’? Is someone being excluded because of it? Does the definition include all groups?

Question 5 *Can the ‘problem’ be thought about differently?*

· What effects does stigma have on the problematization of TB?

· How is stigma used to describe the outcomes (target populations)?

· Does the use of stigma for the problematization of TB cause unintended exclusion?

Question 6 *What effects are produced by this representation of the ‘problem’?*

· Would the inclusion of stigma enable the policy to target a wider population affected by TB and TB/HIV?

· Are the outcomes of the policy stigma specific? How can this be adapted to be more stigma inclusive

**SM 2: Appendix B Analysis Based on the WPR Approach**

|  | | **Portugal** | **Brazil** | **Mozambique** | **Belarus** | **Romania** | **Indonesia** | **Netherlands** |
| --- | --- | --- | --- | --- | --- | --- | --- | --- |
| **Definition of TB** | | An infectious disease (medical definition). | An infectious disease, characterized as a chronic condition with a strong social determination and a barrier to socio-economic development. | An infectious disease that is a threat and an enemy to the public health of the Mozambican population. | An infectious disease  (medical definition). | An infectious disease  (medical definition). | An infectious disease  (medical definition). | An infectious disease (medical definition). |
| **Stigma** | **Definition** | Not defined but mentioned. | Conceptualized as a barrier to end TB and related with human rights and equity in health [BR1, BR2, BR3]. | Not defined or mentioned. | Not defined or mentioned. | Not defined but mentioned. | Not defined but mentioned. | Not defined or mentioned, but a stigma study is referenced |
|  | **Locating responsibility for being stigmatized** | Structural Responsibility:  improvement of TB literacy for the population and healthcare professionals.    Individual Responsibility:  use of stigmatizing language which implies that being cured is the responsibility of the person affected by TB. | Structural Responsibility:  recognition of systemic discrimination (based on race, sex, gender) that can increase the TB stigma associated with certain groups. | Individual Responsibility: use of stigmatizing language which implies that being cured is the responsibility of the person affected by TB. | Structural Responsibility:  increasing emotional support and psychological assistance to reduce individualized stigma,  measures to educate healthcare professionals for anti-discrimination.    Individual Responsibility: stereotypes the individual with TB.  lack of population education (measures taken), use of stigmatizing language. | Structural Responsibility: educate the population, provide psychosocial services, attempting to ensure anti-discrimination.  Individual Responsibility: lack of population education (measures taken), use of stigmatizing language.  Determination of being cured is the responsibility of the person affected by TB | Structural Responsibility:  educate the population, and healthcare professionals, engage the community in promoting an environment free of stigma and discrimination for people and family members living with TB.    Individual Responsibility:  stigma is seen as a reason for the individual not seeking healthcare and therefore causing the underreporting of TB. | Individual responsibility:  people are discriminated against due to correlation with migrant status.  "tuberculosis in the Netherlands is closely linked to the inflow of immigrants and asylum-seekers… " [3 |
|  | **Language** | No use of stigmatizing language. | Emphasis on the humanity of the disease - not recurrent use of stigmatizing language. | Use of stigmatizing language:  ‘Advise all healthcare workers in contact with  infectious patients to use a respirator (N95) for  personal protection’ [M1, p.34]. | Use of stigmatizing language:  ‘*dangerous’*  *‘‘Increased epidemiological threat.’* [BE1, p.4]. | Use of stigmatizing language:  ‘*Identification of TB suspects*’ [R1 p. 3]. | Use of stigmatizing language:  ‘*Develop TB suspect referral tools* [...]’ [I1, p. 196]. | No use of stigmatizing language. |
| **Social Inequities** | **Problematization** | Social inequities are neglected – except for the acknowledgment of geographic disparities.  Problematization as a narrow biomedical public health problem - focus on diagnosis and treatment, constant use of statistical data. | Social inequities are acknowledged by need to address socio-cultural practices, education and health system deficiencies them.  Problematization as a narrow biomedical public health problem - their documents are the result of multidisciplinary work, with equal focus on diagnosis, treatment, and prevention [BR1, BR2, BR3]. | Social inequities are framed as epidemiological risk factor.  Problematization as a biomedical public health problem - focus on diagnosis and treatment, and constant use of statistical data.. | Social inequities are acknowledged by need for multisectoral and interdepartmental approaches to health, geographic disparities, and development of primary healthcare.  Problematization as a biomedical problem, and through identification of risk groups, social construction of TB can be implied through the statistical patterns identified.  However, the National Programme for Health identifies the need for multisectoral and interdepartmental approaches to health including TB. | Social inequities framed as epidemiological risk factor.  Problematization as a biomedical problem, risks-groups are identified through geographic distribution of TB and based on statistical patterns. | Social inequities acknowledged by need to address social protection deficiency.  Main targets include improving health equity. | Social inequities framed as an epidemiological risk factor.  Problematization as a biomedical problem, risks-groups are identified through geographic distribution of TB and based on statistical patterns |
|  | **Measures taken** | Focus on early diagnosis and treatment (disease control) for vulnerable populations.    Emphasis on vaccination and TB screening for children of vulnerable groups.    Screening professionals with high-incidence jobs.    Education. | Strategic actions aimed at the populations most vulnerable to falling ill with TB and availability of incentives, social assistance, transportation, etc.  Prioritization of TB control and prevention.  Systematic screening (independent of who they are).  Education. | Biomedical measures (vaccine, prophylaxis) related to prevention, not tackling socio economic problems.  Active search of cases, through ‘*Cough Officials’* [M1, p.33]. | Measures include providing prevention, diagnosis, and treatment of TB for vulnerable populations.    Prophylactic treatment for people with presumptive TB and contacts.    Social support is provided in the form of convenience for people living with TB to access treatment, social and material assistance. The underlying aim of it is to increase adherence to treatment.    Letter to population calling for reporting of people with TB symptoms. Health professionals on the lookout for people with TB symptoms.    Involuntary hospitalization of people living with TB evading treatment, court mandated treatment. | DOT strategy, but there is a gap in implementation as it was inconsistent and vulnerable populations have limited access.    Social support is provided to increase adherence to treatment (incentives).    Screening of inmates upon entry to prison.    The person affected by TB can refuse treatment, but then social support is removed, and Penal Code punishes knowingly infecting other people. | Increase social and community participation.    Active case finding, especially in at-risk groups (but also at general public) and maximize contact investigation activities.    Develop a referral system for TB suspects.    Support services and social protection for people reported as TB cases (no specification.    Expansion of the DOTS services.    Improve and expand educational campaigns. | Active case finding in new immigrants and asylum-seekers from high TB-burden countries through enforced screening. |
|  | **Allocation of responsibility** | Individual Responsibility:  certain groups do not seek healthcare services. | Structural Responsibility: the municipalities, should act according to the national guides but adapt the measures to be implemented to their population; and the health care personal should know how to identify barriers outside the medical field. | Structural Responsibility: recognition of structural problems related to the healthcare system (lack of material or diagnostic equipment).    **I**ndividual Responsibility: people are responsible for bad adherence, their social conditions and may be punished for it. | Structural Responsibility: government responsibility to carry out measures to strengthen the treatment of the people living with the TB (not further specified).    Individual Responsibility: regarding treatment. Emphasis on “*patient motivation is critical*”. Focus on “*bad habits of the population*” as the reason for active spread. | Structural Responsibility: facilities deemed high risk are responsible to develop and implement their own TB transmission control plan.    Individual Responsibility: for adherence to treatment. | Structural Responsibility:  decentralization of the healthcare system, making the district responsible to achieve the proposed goals; and strengthen methods of diagnostic and treatment. | Individual Responsibility:  social inequalities not mentioned. Responsibility for infection is being an Immigrant or asylum seeker |
| **Gender** | **Definitions/Framing** | Is not defined, however is presented related to TB in epidemiological and statistical terms. | | | Defined by sex. | Defined by sex, but the policies are rather gender neutral. | Defined by biological sex. | Not defined. |
|  | **Gender as Binary** | ‘*women’* and ‘*men’,* ‘*female’* and ‘*male’* | Gender-neutral terms – people affected by TB (progressive, but not inclusive). | ‘*women’* and ‘*men’, female’* and ‘*male’* | ‘*women’* and ‘*men’* | ‘*women’* and ‘*men’,* ‘*female’ and* ‘*male’ b*ut neutral in policy targets. | ‘*women’* and ‘*men’*, but neutral in policy targets. | Gender-neutral terms – ‘people with TB’, ‘patients’ ‘asylum seekers’ ‘immigrants’ |
|  | **Gender as Risk Group** | Use of statistical and demographic data to justify being ‘*male’* and a ‘*pregnant woman*’ as a risk group. | | | Men are not mentioned as a risk group in this case. Women are identified as a risk group and targeted for prevention and treatment policies. | An epidemiological relationship is identified, with men accounting for most of the TB cases, but no specific targets or outcomes are set out to focus on men as a risk group. | Neither men or women are mentioned explicitly as a risk group. | Men are implied as being ‘*at risk’* due to the higher proportion being immigrants, asylum  seekers, or detainees. |
|  | **Focus on Women of Reproductive Age** | No. | Pregnant woman are a focus group – effects on the fetus in case of TB disease. | Pregnant woman, women in the ‘childbearing phase’ and ‘lactating woman’ as a focus group – effects on the fetus in case of TB disease and discussion of sex education. | Problem is represented to be through the reproductive function of the female sex. “Women of reproductive age”  “Preservation or elimination of specific female functions (menstrual, childbearing, etc.)”, with a focus on the fertility rate in the country [BE2]. | No. | No. | No. |
